# Efficient Transformation of Water Vapor into Hydrogen by Dielectric Barrier Discharge Loaded with Bamboo Carbon Bed Structured by Fibrous Material

**DOI:** 10.3390/molecules29143273

**Published:** 2024-07-11

**Authors:** Hui Xu, Ran Sun, Yujie Tan, Chenxiao Pei, Ruchen Shu, Lijie Song, Ruina Zhang, Chuang Ouyang, Min Xia, Jianyuan Hou, Xinzhong Zhang, Yuan Yuan, Renxi Zhang

**Affiliations:** 1 Institute of Environmental Science, Fudan University, Shanghai 200433, China; xuhui_2021@163.com (H.X.); 23110740020@m.fudan.edu.cn (R.S.); 20210740088@fudan.edu.cn (Y.T.); 22210740066@m.fudan.edu.cn (C.P.); 21210740080@m.fudan.edu.cn (R.S.); hjy@fudan.edu.cn (J.H.); zhangxinzhong@fudan.edu.cn (X.Z.); yuan_y@fudan.edu.cn (Y.Y.); 2 Shanghai Institute for Design & Research on Environmental Engineering, Shanghai 200232, China; zhangrn@huanke.com.cn (R.Z.); ouyc@huanke.com.cn (C.O.); xiam@huanke.com.cn (M.X.)

**Keywords:** dielectric barrier discharge, argon, water vapor, hydrogen, bamboo carbon, free radical reactions

## Abstract

A new method of efficiently transforming water vapor into hydrogen was investigated by dielectric barrier discharge (DBD) loaded with bamboo carbon bed structured by fibrous material in an argon medium. Hydrogen productivity was measured in three different reactors: a non-loaded DBD (N-DBD), a bamboo carbon (BC) bed DBD (BC-DBD), and a quartz wool (QW)-loaded BC DBD (QC-DBD). The effects of the quality ratio of BC to QW and relative humidity on hydrogen productivity were also investigated in QC-DBD at various flow rates. The reaction process and mechanism were analyzed by scanning electron microscopy, X-ray photoelectron spectroscopy, N_2_ physisorption experiments, infrared spectroscopy, and optical emission spectroscopy. A new reaction pathway was developed by loading BC into the fibrous structured material to activate the reaction molecules and capture the O-containing groups in the DBD reactor. A hydrogen productivity of 17.3 g/kWh was achieved at an applied voltage of 5 kV, flow rate of 4 L/min, and 100% relative humidity (RH) in the QC-DBD with a quality ratio of BC to QW of 3.0.

## 1. Introduction

Traditional fossil energy sources are non-renewable, and their combustion products are polluting. Hydrogen is considered to play an important role in providing carbon-free energy [1], and efficient hydrogen energy systems could reduce the dependence on functional fossil fuels for heating, transportation, industry, and electricity [2,3]. Existing technologies for hydrogen generation include water electrolysis [4,5], ultraviolet (UV) light [6,7], biomass pyrolysis [8], and ionizing radiation [9]. Among the above methods, water electrolysis has attracted much attention and is considered to be the most promising method for hydrogen production [10]. Despite the significant advantages of electrochemical water decomposition, most efficient hydrogen production methods still require durable precious metal catalysts to facilitate reaction kinetics [11]. However, low precious metal reserves and high costs have limited the industrial application of precious metal catalysts in electrochemical water decomposition [12]. Alkaline electrolysis systems do not require the use of precious metal catalysts, but they need to be operated at high efficiency, low current density, and low operating pressure [13], which increases the size of the system and makes them unsuitable for frequent startups and variable power inputs [14]. The catalytic low-cost and easy-to-operate electrochemical decomposition of water for hydrogen production deserves further exploration.

Because the electrochemical decomposition of water for hydrogen production by dielectric barrier discharge (DBD) has high energy density at atmospheric pressure and room temperature [15], it can generate a lot of energy in a small space, which is advantageous for compact energy storage or conversion systems. Hydrogen production by DBD is potentially viable, especially in portable mobile scenarios. Owing to its simplicity, rapidity, and cleanliness, the transformation of water vapor into hydrogen using a DBD reactor is considered a promising green method for hydrogen production with easy access, simple geometry, and instantaneous production [16,17].

Varne et al. [16] evaluated the optimal conditions for hydrogen production from an argon–water dielectric barrier discharge. Muhammad et al. [18] used an optimized corona–DBD hybrid plasma microreactor to produce hydrogen from water vapor. However, the impact of oxidizing atmospheres enriched with O and H_2_O_2_ consequently affects the production of H atoms as precursors to H_2_ under pure argon–water discharge conditions [19]. Given the significant influence of O functional groups on hydrogen production, it is imperative to establish an oxygen-free environment. Carbonaceous materials have a high surface area [20], and due to the high affinity of carbon for oxygen, carbon reacts with elemental oxygen, contributing to the positive water-to-hydrogen reaction [21,22]. Non-thermal plasma-enhanced carbon excitation can lead to a synergistic effect that induces the reaction to occur [23,24]. Since bamboo carbon (BC) is the simplest and most readily available reducing substances, by using bamboo carbon, it might be possible to achieve the desired reaction rates with less material, thus reducing the overall amount of catalyst required for the process. Combining DBD and BC to create an oxygen-free atmosphere can be considered a good concept for hydrogen production through water. Relevant studies and mechanistic investigations have not been reported.

In this study, an innovative approach was investigated for the conversion of water vapor into high-yield hydrogen at ambient temperatures and pressures by a DBD loaded with a bamboo carbon bed structured with fibrous materials. Activated carbon atoms were utilized as trapping agents for oxidizing substances. H_2_ productivity in three different reactors with no loaded DBD (N-DBD), bamboo carbon (BC)-packed bed DBD (BC-DBD), and quartz wool (QW)-loaded BC DBD (QC-DBD) were examined. The effects of different discharge voltages, vapor flow rates, quality ratios of BC to QW, and relative humidities on hydrogen yields were also explored. The packed materials were characterized in detail, both physically and chemically, before and after use in the DBD reactor to reveal the reaction process and mechanism.

## 2. Results and Discussion

### 2.1. The Characterization of the Packed Materials in QC-DBD

#### 2.1.1. Morphology

**Figure 1:**
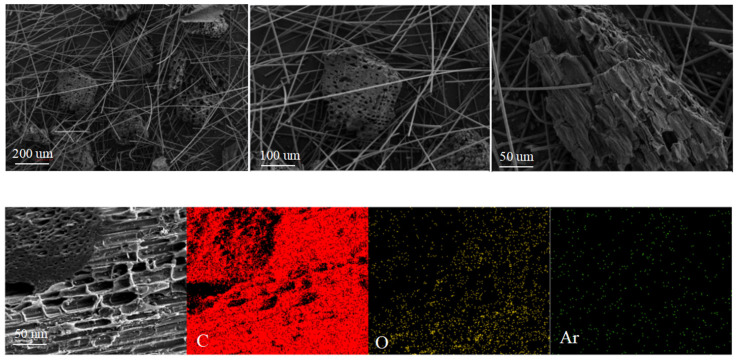
SEM-EDS images of BC-Used in QC-DBD.

Figure 1 presents the SEM images of the BC loaded on the QW, showing that the BC was wrapped by a large number of QW filaments. The introduction of QW led to the adsorption of charged particles, which in turn facilitated the generation of hydrogen on the BC within the QC-DBD framework. From the EDS elemental mapping results of BC-Used, a certain amount of Ar atoms was observed on the surface of the BC. The observed phenomenon provides evidence of electron transfer between the excited Ar atoms and the BC substrate. Simultaneously, the significant amounts of oxygen attached to the BC surface indicated the successful capture of oxygen-containing functional groups from water by the activated carbon atoms.

#### 2.1.2. BET Results

**Figure 2:**
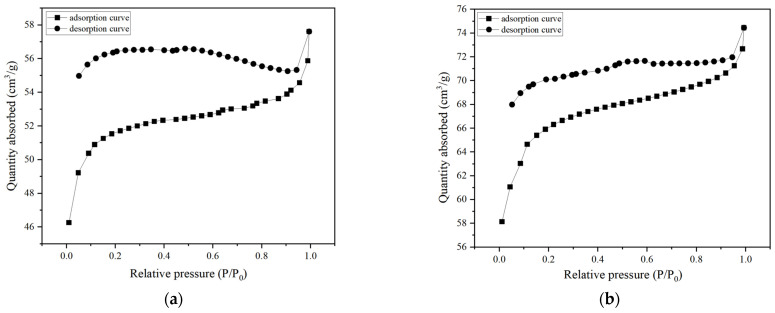
Nitrogen adsorption/desorption isotherms and the corresponding pore size distribution curves (inset) of (**a**) BC-Before and (**b**) BC-Used.

The N_2_ adsorption and desorption isotherms and pore size distribution (PSD) curves of BC were analyzed, and the textural properties of BC are discussed. Figure 2 shows that BC has characteristic Type IV isotherms of the Bruno–Demin–Taylor classification [25], indicating the presence of mesoporous/microporous structures. According to the PSD curves, the pore sizes of the two catalysts are similar, mainly containing small mesopores (<10 nm) and micropores (<2 nm). The pore size distribution of BC was calculated using the N_2_ adsorption isotherm and the Barrett–Joyner–Halenda (BJH) adsorption branch. The persistence of the natural BC structure after plasma excitation was evidenced by the presence of a similar hysteresis loop. Compared with BC-Before, BC after plasma excitation exhibited a higher degree of mesoporous structures. These newly created pores contribute to an increase in the specific surface area (S_BET_), as shown in Table 1.

#### 2.1.3. XPS Characterization

**Figure 3:**
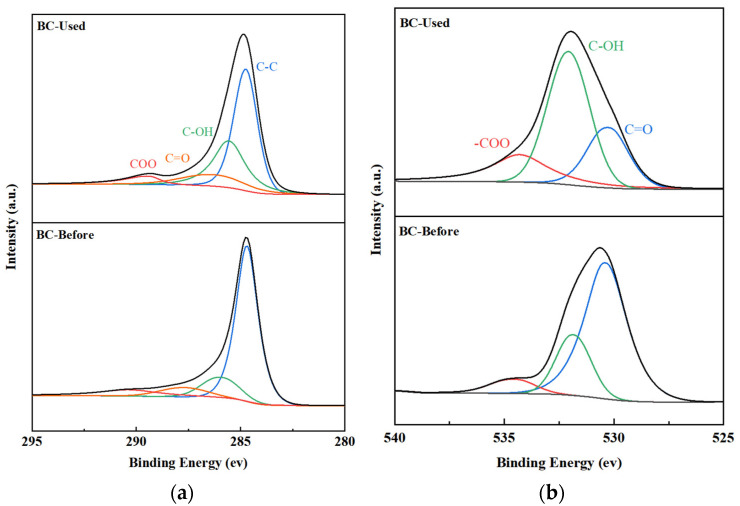
C 1s and O 1s XPS signals of (**a**) BC-Before and (**b**) BC-Used.

Table 1 lists the surface chemical states of BC as determined by XPS. The surface oxygen content of BC-Used was significantly higher than that of BC-Before. Figure 3a presents the C 1s spectrum of the BC, which can be assigned to the following bands: 284.8 eV for C-C, 286.1 eV for the carbon bound to oxygen alone (i.e., C-OH) in phenol and ether, 287.5 eV for the carbon doubly bound to oxygen in ketone and quinone (i.e., C=O), and 288.7 for the carbon bound to both oxygen (i.e., -COO) in carboxylic anhydride and ester eV [26]. The generation of a greater number of C-based functional groups in BC-Used provides clear evidence for the participation of BC in water vapor conversion reactions. The O1s XPS spectrum of the sample is shown in Figure 3b. All O1s spectra can be assigned here: C=O at 531.2 eV, C-O at 533.2 eV, and -COO at 534.8 [27,28]. The surface-bound oxygen species, including C-O and OH groups, were prone to instability during the reaction process. Hydrogenation led to an increase in the number of reactive oxygen species on the BC surface.

#### 2.1.4. FT-IR Analysis

**Figure 4:**
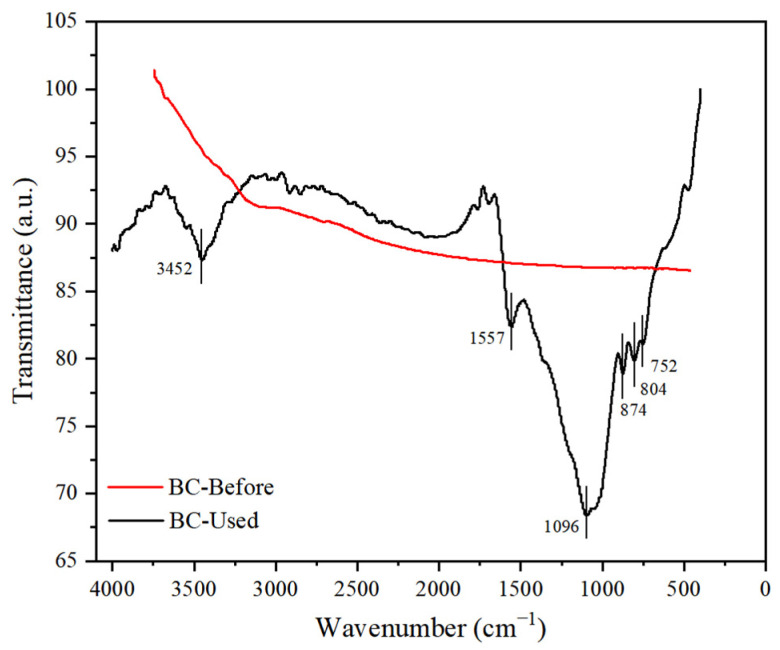
Fourier transform infrared spectra of BC-Before and BC-Used.

As shown in Figure 4, FT-IR spectroscopy was used to characterize the functional groups in the BC. For the BC-Before surface, no obvious bands were observed in the spectra, suggesting that the functional groups on the BC surface were formed by the intra-DBD reaction after use. For BC-Used, a strong band at ~1096 cm^−1^, along with those at ~ 752, 804, 874, 1557, and 3452 cm^−1^, was observed. The bands at ca. 3450 and 1557 cm^−1^ were assigned to the stretching and bending vibrations of the O-H bond, respectively [29]. The bands from ca. 1700 to 1760 cm^−1^ correspond to C=O stretching vibrations in carboxylic acids, and those at 1096 cm^−1^ correspond to the epoxy (C-O-C) and alkoxy (C-OH) groups on the BC surface, respectively [30]. Owing to the addition of QW in the DBD reaction, Si-O also existed around ca. 1100 cm^−1^, and the peak at around ca. 700–900 cm^−1^ may indicate the existence of Si-C. The above characterization of BC-Used provides a potential explanation for the ability of BC to facilitate hydrogen production by capturing oxygen-containing functional groups from water.

#### 2.1.5. Optical Emission Spectroscopy

**Figure 5:**
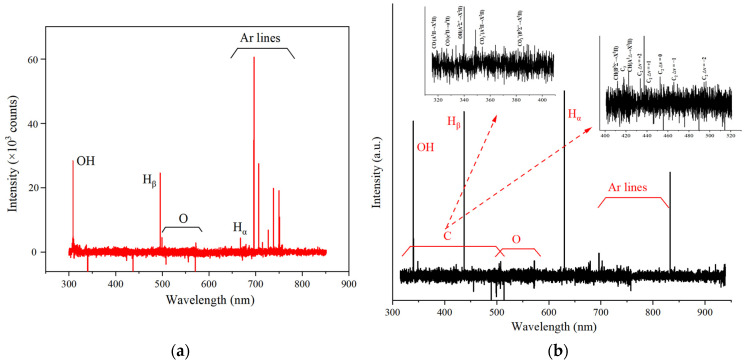
OES spectrum observed in (**a**) N-DBD and (**b**) QC-DBD (reaction conditions: 100% RH; BC: QW quality ratios: 3.0; flow rate: 0.5 L/min; discharge voltage: 5 kV).

The plasma environment provides high-energy electrons, ions, excited species from background gas molecules, and free radicals from reactants [31]. From the spectra shown in Figure 5a, the emission spectra of OH, O, H_α_, H_β_, and Ar generated by these background gasses are detected in N-DBD. QC-DBD, as shown in Figure 5b, exhibited more obvious excitation spectra of C atoms, while the intensity of O was reduced, and the emission spectra of CO and CO_2_^+^ were observed. Moreover, the intensities of OH and H were much stronger than those in the reaction without the addition of BC, which explains the enhanced H_2_ production.

### 2.2. Experimental Results and Analysis

#### 2.2.1. Effects of Discharge Voltage on H_2_ Productivity in Different DBD Reactors

**Figure 6:**
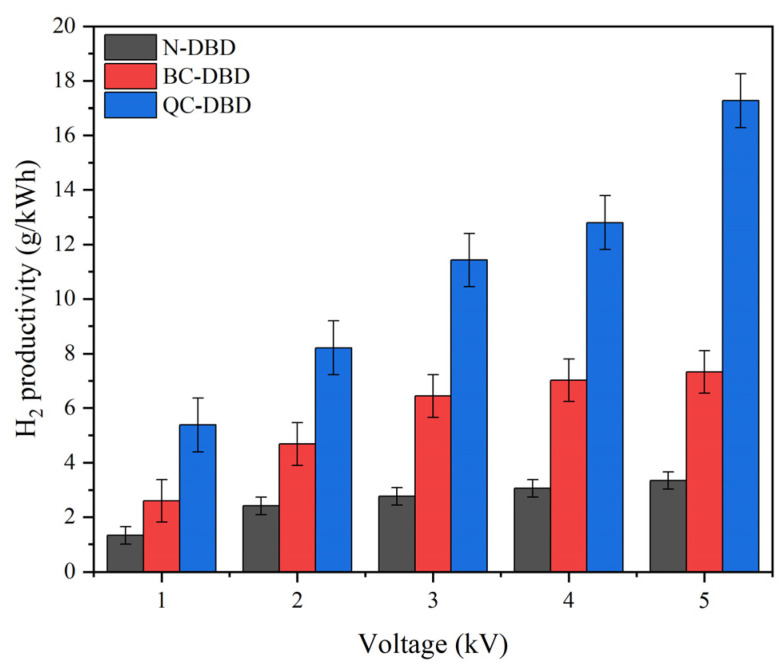
H_2_ productivity under different DBD reactors at various discharge voltages (reaction conditions: 100% RH; flow rate: 4 L/min; mass of BC: 0.15 g in BC/QC-DBD).

In Figure 6, the H_2_ productivity in different DBD reactors is presented at applied voltages varying from 1 to 5 kV. The results showed that while the H_2_ productivity increased with the applied voltage, an obvious enhancement occurred in the QC-DBD, and the productivity remained almost steady in the N-DBD. The best H_2_ productivity in QC-DBD reached 17.3 g/kWh at the voltage of 5 kV, while it was only 7.3 g/kWh and 3.3 g/kWh in BC-DBD and N-DBD, respectively.

Furthermore, H_2_ productivity was higher in BC-DBD and QC-DBD than in N-DBD at similar voltages. The main reason for this is that even at ambient temperature and pressure, the carbon atoms excited by high-energy electrons may lead to the reaction C + H_2_O → CO + CO_2_ + H_2_. The presence of activated carbon atoms is conducive to creating a reducing atmosphere for the system by capturing oxygen-containing functional groups and promoting the reaction process with an increase in the micro-discharge process. Meanwhile, the discharge pattern varied from a simple filament discharge to a complex discharge involving micro-discharge among the carbon particles [32].

In QC-DBD, the structural properties of the fibrous material lead to changes in the electric field structure, contributing to increased electronic activity. During the diffusion of the reactants to the QC surface, some H_2_O and carbon atoms are activated by the discharge plasma and enter the reactive vibrational excited state or even the dissociated state. Then, the fibrous material is more likely to gather a large amount of charge at the tip of the fiber, leading to a higher probability of electron collision, which is beneficial for the transformation of water vapor into hydrogen. Meanwhile, the temperature in QC-DBD was significantly lower than that in BC-DBD, mainly because the aggregation of BC caused thermal accumulation [23], which hindered heat dissipation as the voltage increased. While the discharge space among the BC particles was expanded by loading on the fibrous material, there was better heat dissipation and enhanced micro-discharge capability.

The transformation of water vapor into hydrogen in QC-DBD was compared with previously published results. As displayed in Table 2, a hydrogen productivity of 17.3 g/kWh was achieved in this work at a flow rate of 4 L/min and an applied voltage of 5 kV, which was significantly higher than that in other studies, except that it was relatively lower than the electrolysis of water. This comparison demonstrates the excellent hydrogen productivity in the QC-DBD reactor.

#### 2.2.2. Effects of the BC: QW Quality Ratios on H_2_ Productivity in QC-DBD

**Figure 7:**
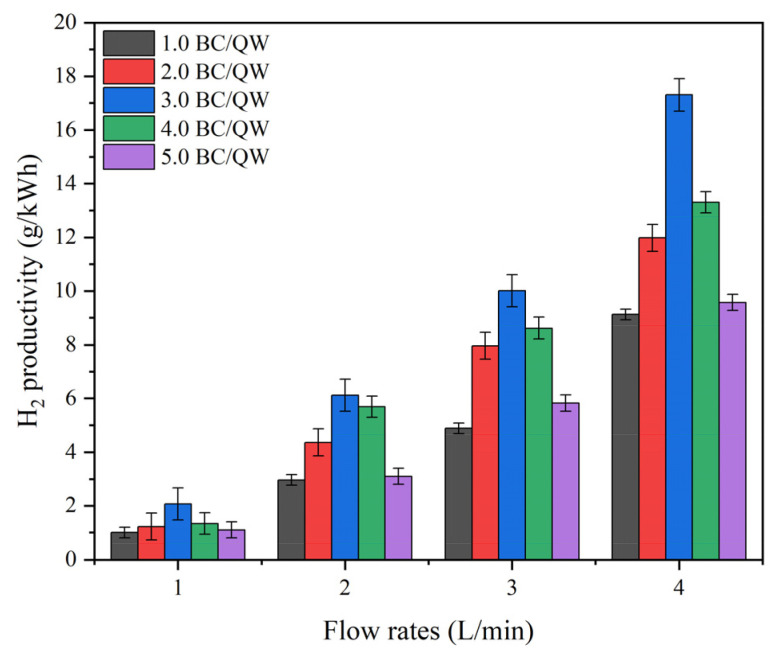
H_2_ productivity under different BC: QW quality ratios in QC-DBD at various vapor flow rates (reaction conditions: 100% RH, discharge voltage: 5 kV).

Another factor affecting the discharge is the particle gap. For a packed bed composed of BC particles and quartz wool (QW), the particle gap can be adjusted indirectly by changing the quality ratio of BC to QW in QC-DBD. Figure 7 shows the effect of different BC-to-QW quality ratios on hydrogen productivity at various vapor flow rates. At a certain flow rate, a quality ratio of BC to QW of 3.0 demonstrated the most favorable effect on hydrogen productivity.

According to Parson’s law, the breakdown voltage v_s_ in a uniform electric field is a function of the product of the gas pressure P and discharge gap d [23]. When the mass of BC increased, the d between the activated carbon particles tended to decrease, and the breakdown voltage v_s_ was larger at this time, thus blocking the discharge phenomenon. Additionally, because BC has more filamentous fibers or tubular structures, most of the electrons enter the BC structure through the channel and are gradually dissipated; electrons are rarely reflected on the sidewalls of the channel [40], leading to poor discharge performance when the quality ratio of BC to QW is high. Conversely, when the BC: QW quality ratio was low, the gap between the BC particles increased. The probability of electron collisions between carbon atoms decreases with a diminished ability to capture oxygen-containing functional groups, which affects hydrogen productivity. At a BC-to-QW quality ratio of 3.0, the most suitable particle gap in the QC-DBD resulted in the best discharge state and a reducing atmosphere.

In addition, a higher flow rate was conducive to higher hydrogen productivity, owing to the increase in water vapor per unit time and the higher probability of electron collisions. It can be concluded that the reactor exhibited an excellent ability to convert water into hydrogen with an instantaneous response.

#### 2.2.3. Effects of Relative Humidity on H_2_ Productivity in N-DBD and QC-DBD

**Figure 8:**
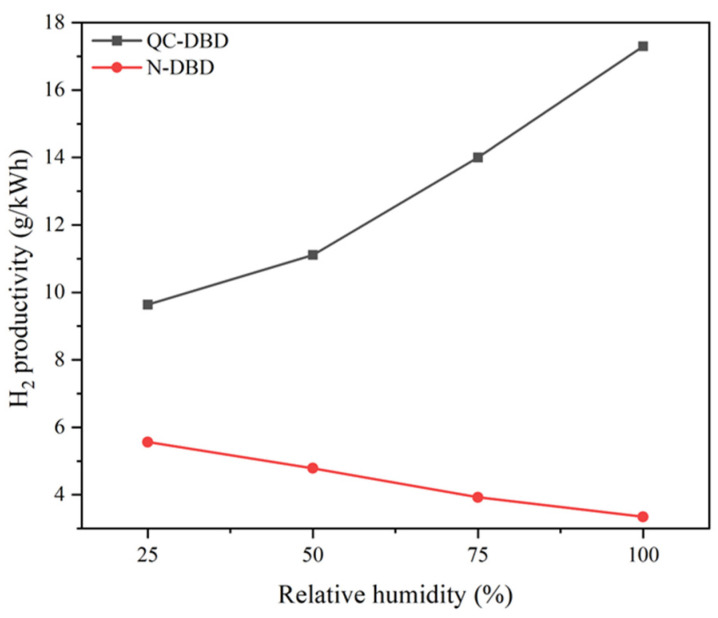
H_2_ productivity under different relative humidity in N-DBD and QC-DBD (reaction conditions: BC: QW quality ratios: 3.0; flow rate: 4 L/min; discharge voltage: 5 kV).

The H_2_ productivity curve of QC-DBD with varying relative humidity exhibited a trend opposite to that of N-DBD, as depicted in Figure 8. These results suggest that the addition of QW improved the humidity tolerance of the QC-DBD reaction system because the gas breakdown voltage threshold may not be the limiting factor affecting H_2_ productivity, although it was altered by water vapor. Additionally, BC with large pores and wrinkled surfaces has a high water absorption capacity when irradiated, which would be beneficial for higher intensity, making the electron movement more intense [41]. Simultaneously, the accumulation of charges on the surface of the QW generates alternating electric fields owing to the fiber structure adsorbing charged particles in the DBD. During the discharge process, these charges undergo repeated aggregation and emission, interacting with the BC to consume a large amount of water vapor to generate hydrogen gas. However, in N-DBD, the highest hydrogen productivity was attained at 25% RH without water vapor buffering by the packing material because of the increase in the gas breakdown voltage threshold with increasing water vapor [16].

#### 2.2.4. Production of CO, CO_2_ and CH_4_ in QC-DBD

**Figure 9:**
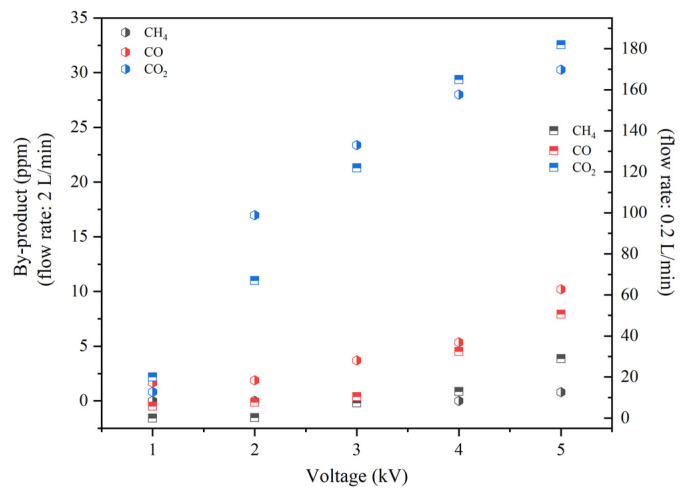
Production of CO, CO_2_, and CH_4_ in QC-DBD (reaction conditions: BC: QW quality ratios: 3.0; flow rate: 0.2 L/min and 2 L/min; 100% RH).

In Figure 9, as the voltage increases, the by-products are also increased; among them, the production of CO_2_ is the highest. When the voltage is higher, slight levels of CH_4_ and CO are achieved with no more than 30 ppm and 70 ppm, respectively. There is a yield of CO_2_, CO, and CH_4_ when mixed with H_2_, which can be used as combustible gases in conjunction with H_2_ or purified through subsequent separation processes.

## 3. Experimental Section

### 3.1. Packed Materials Preparation and Characterization

Bamboo carbon and quartz wool were purchased from Heatton Environmental Technology, Ltd. (Shanghai, China). The bamboo carbon samples were ground and mechanically sieved to obtain particles with a size distribution of 1.0–1.5 mm (30–45 mesh), and then washed thrice using deionized water to remove impurities and dried at 100 °C for 12 h. To prepare the quartz-wool-loaded bamboo carbon powder reductant, different quality ratios of bamboo carbon to quartz wool were mixed with 10 mL of ethanol solution in five beakers. The beakers were placed in an oven at 100 °C for 12 h until the ethanol solution completely evaporated.

BET surface area measurements were conducted on a 4-station fully automated specific surface area analyzer: Micromeritics APSP Model 2460 (Micromeritics, Norcross, GA, USA). Prior to the BET measurements, 0.1 g of catalyst was placed in a U-reactor and purged with flowing helium at 110 °C.

Surface microstructure analysis was performed using an FEI-NOVA NANOSEM 230 (EDS X-MAX50) (FEI, Hillsboro, OR, USA) SEM at an accelerating voltage of 20 kV to examine the surface characteristics of the three samples. Energy-dispersive X-ray spectroscopy (EDS) was employed to examine the content and distribution of surface elements.

X-ray photoelectron spectroscopy (XPS) was conducted using a Thermo Scientific K-Alpha instrument (Thermo Fisher Scientific, East Grinstead, UK) with Al K-alpha radiation and penetration energies of 20–40 eV. To account for the effect of the surface charge, the binding energy was corrected with reference to C 1s (284.8 eV).

Fourier-transform infrared (FT-IR) spectroscopy was performed using an IR-Tracer-100 instrument (Shimadzu, Kyoto, Japan) with KBr as the reference material.

Optical emission spectroscopy (OES) was used to observe the emission spectra of the DBD reactor, which was purchased from AVANTES (Shanghai, China).

Gas chromatographs (GC-9860E, Linghua Instrument, Shanghai, China) were utilized to detect hydrogen production and CO, CH_4_, and CO_2_ content in the outlet gas. The setup utilized a TDX-01 packed column (Jingke Rui Da, Beijing, China), with a TCD (Thermal Conductivity Detector) as the detector, nitrogen as the carrier gas, a filament current of 80 mA, a column temperature set to 70 °C, a detector temperature of 80 °C, and an injector temperature of 90 °C.

### 3.2. Experimental Setup

**Figure 10:**
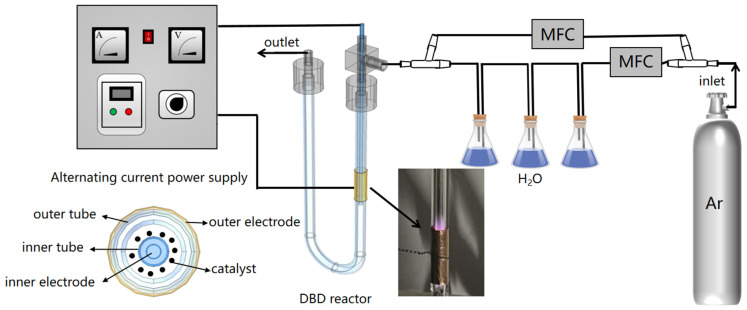
Diagram of the experimental arrangement.

The device used for the hydrogen production reaction is depicted in Figure 10 and consists of a continuous-flow gas generation system, a reaction system, and outlet gas analysis. Ar gas (99.99% purity) was directed into two separate paths using a three-way pipe. One branch was directly connected to the DBD reactor, while the other facilitated the passage of argon gas through three sealed flasks filled with distilled water to obtain saturated argon water. A Horiba Stec-4400 mass flow controller (HORIBA, Kyoto, Japan) was utilized for independent control of the inlet flow rate on both branches, enabling the adjustment of different percentages of water vapor in the argon gas and the gas flow rate before entering the DBD reactor. Prior to entering the DBD reactor, the humidity levels were measured using a psychrometer (Testo 605-H1, Testo SE & Co. KGaA, Titisee-Neustadt, Germany). The DBD reactor was connected to high-voltage AC power. The power was analyzed using a digital oscilloscope (TDS2024B, Tektronix, Beaverton, OR, USA). AC was placed in the DBD reactor, including an inner tube (quartz glass, 11 mm outer diameter, 275 mm tube length, 1 mm wall thickness), an outer tube (quartz glass, 21 mm inner diameter), an inner electrode (stainless steel sheet wrapping the inner wall of the inner tube, 0.3 mm thickness, 165 mm length), and an outer electrode (copper sheet, 20 mm wide). The temperature outside the reaction zone walls was monitored and recorded using an infrared thermometer (Testo 830-T1, Testo SE & Co. KGaA, Germany).

The formula for the hydrogen productivity is shown in Equation (1).

(1) $$H_{2}\mathrm{productivity}\;(g/\mathrm{kWh})\;=\;\left(C\times 2/22.4\right)\times \left(Q\times 60/10^{6}\right)/\left(D^{2}\times \pi /Q/1000\times 60\right)/3600/P_{2}\times 1000$$

where *C* is utilized to denote the concentration of a substance in parts per million in ppm, *Q* is the total flow rate in mL/min (gas volume was measured at room temperature, ∼25 °C), *D* is the radius of the discharge zone in cm, and $P_{2}$ is the true discharge power in w.

## 4. Reaction Mechanism

In QC-DBD, BC loading onto the fibrous-structured material prevents the reformation of H_2_O in the reaction and promotes H_2_ production by capturing O-containing groups. Peaks of C-OH and C=O were found in the FT-IR spectrum of BC-Used, indicating that O and OH bind to the excited C atoms. Combined with the above BC-Used characterization and OES results, the various reactions occurring in QC-DBD responsible for H_2_ formation are as follows:

Carrier Ar plasma discharge generates Ar*, Ar^+^, etc.

(2) $$e^{-}+\mathrm{Ar}\underset{\;}{\to }{\mathrm{Ar}}^{*}+e^{-}\underset{\;}{\to }{\mathrm{Ar}}^{+}+2e^{-}$$

Packed-bed BC plasma discharge generates C*, etc.

(3) $$e^{-}+C\underset{\;}{\to }C^{*}+e^{-}$$

(4) $${\mathrm{Ar}}^{*}+C\underset{\;}{\to }C^{*}+\mathrm{Ar}$$

These interact with water vapor through reactions (5)–(7).

(5) $${\mathrm{Ar}}^{*}+H_{2}O\underset{\;}{\to }H_{2}O^{*}+\mathrm{Ar}$$

(6) $$e^{-}+H_{2}O\underset{\;}{\to }H_{2}O^{*}+e^{-}$$

(7) H2O*→ H. +O.H

The O-containing groups are captured by C^*^.

(8) O .H+C*→ C .OH→ CO+CO2+CH4

(9) C .OH+e−→ C .O+C .OO+C .H→ CO+CO2+H2

H^.^ dimerizes to H_2_

(10) $$H^{.}+H^{.}\underset{\;}{\to }H_{2}$$

Compared with the final products in N-DBD, there was no H_2_O_2_ or O_3_ in QC-DBD. These results indicate that the addition of BC to the packed bed of DBD creates a well-reducing atmosphere and promotes the reaction process of H_2_ production.

A fraction of the by-products is consumed through reactions (11) and (12).

(11) H.+C*→ C .H→ CH4

(12) $${\mathrm{CH}}_{4}+{\mathrm{CO}}_{2}\underset{\;}{\to }\mathrm{CO}+H_{2}$$

**Figure 11:**
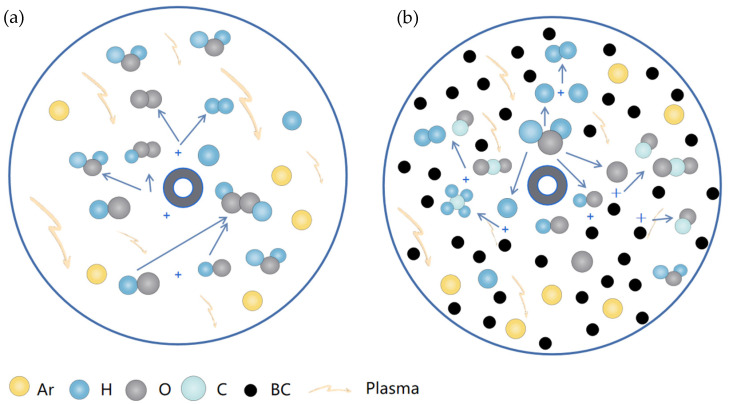

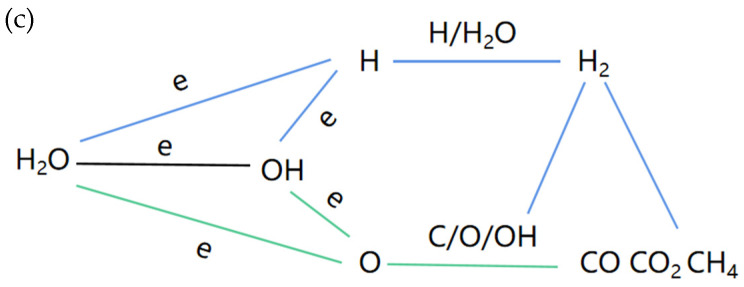
Reaction mechanisms in (**a**) N-DBD-, (**b**) QC-DBD-, and (**c**) QC-DBD-assisted water splitting.

The main reactions for H_2_ production in N-DBD and QC-DBD are shown in Figure 11.

## 5. Conclusions

Hydrogen productivity was investigated at various discharge voltages in three different reactors of N-DBD, BC-DBD, and QC-DBD. QC-DBD was found to be the most efficient hydrogen generator. This study revealed that a specific quality ratio of BC (bamboo carbon) to QW (quartz wool), precisely 3.0, exerted the most advantageous impact on the hydrogen productivity within the QC-DBD reactor. The fibrous nature of the material within the reactor was observed to accumulate a substantial charge, thereby enhancing the likelihood of electron collisions. A noteworthy aspect of the QC-DBD reactor is its exceptional resistance to water; even at a relative humidity of 100%, the reactor sustained high yields of H_2_. The promotion of H_2_ production was suggested to be BC loading onto the fibrous-structured material to activate the reaction molecules and capture O-containing groups in the QC-DBD reactor. A hydrogen productivity of 17.3 g/kWh was achieved at 100% RH, a 4 L/min flow rate, and an applied voltage of 5 kV in the 3.0 QC-DBD. This achievement underscores the potential of the QC-DBD system for efficient and sustainable hydrogen production.

## Figures and Tables

**Table 1:** Surface properties of BC.

BC	S_BET_ (m^2^/g)	d_p_ (nm)	V_p_ (cm^3^/g)	O 1s (%)	C 1s (%)
Before	160.70	2.06	0.08	11.4	88.6
Used	206.79	2.09	0.11	27.9	72.1

**Table 2:** Comparison of the energy yields for different hydrogen production methods.

Production Method	Hydrogen Production Gas	Energy Yield, g(H_2_)/kWh	Reference
Electrolysis	H_2_O	>20	[33]
Photocatalysis	H_2_O	0.01	[34]
AC gliding arc	H_2_O	1.3	[35]
Corona	H_2_O	2.0	[36]
Microwave (2.45 MHz)	C_2_H_5_OH + H_2_O	14.8	[37]
DBD	CH_3_OH + H_2_O	3.3	[38]
	NH_3_	4.1	[39]
	H_2_O	5.5	[16]
DBD coupled BC	H_2_O	17.3	Present work

## Data Availability

Data are contained within the article.
